# 2-Chlorofatty Aldehyde Elicits Endothelial Cell Activation

**DOI:** 10.3389/fphys.2020.00460

**Published:** 2020-05-08

**Authors:** Jane McHowat, Shubha Shakya, David A. Ford

**Affiliations:** ^1^Department of Pathology, Saint Louis University School of Medicine, St. Louis, MO, United States; ^2^Center for Cardiovascular Research, Saint Louis University School of Medicine, St. Louis, MO, United States; ^3^Edward A. Doisy Department of Biochemistry and Molecular Biology, Saint Louis University School of Medicine, St. Louis, MO, United States

**Keywords:** endothelial cells, fatty acids, inflammation, lipid mediators, plasmalogens, vascular biology

## Abstract

Endothelial activation and dysfunction are hallmarks of inflammation. Neutrophil-vascular endothelium interactions have significant effects on vascular wall physiology and pathology. Myeloperoxidase (MPO)-derived products released from activated neutrophils can mediate the inflammatory response and contribute to endothelial dysfunction. 2-Chlorofatty aldehyde (2-ClFALD) is the direct oxidation product of MPO-derived hypochlorous acid (HOCl) targeting plasmalogen phospholipids. The role of 2-ClFALD in endothelial dysfunction is poorly understood and may be dependent on the vascular bed. This study compared the role of 2-ClFALD in eliciting endothelial dysfunction in human coronary artery endothelial cells (HCAEC), human lung microvascular endothelial cells (HLMVEC), and human kidney endothelial cells (HKEC). Profound increases in selectin surface expression as well as ICAM-1 and VCAM-1 surface expression were observed in HCAEC and HLMVEC. The surface expression of these adherence molecules resulted in robust adherence of neutrophils and platelets to 2-ClFALD treated endothelial cells. In contrast to HCAEC and HLMVEC, 2-ClFALD-treated HKEC had substantially reduced adherence molecule surface expression with no resulting increase in platelet adherence. 2-ClFALD-treated HKEC did have an increase in neutrophil adherence. All three endothelial cell lines treated with 2-ClFALD displayed a time-dependent loss of barrier function. Further studies revealed 2-ClHDyA localizes to ER and Golgi when using a synthetic alkyne analog of 2-ClFALD in HCAEC and HLMVEC. These findings indicate 2-ClFALDs promote endothelial cell dysfunction with disparate degrees of responsiveness depending on the vascular bed of origin.

## Introduction

Changes at the blood-endothelial interface can elicit profound changes in organ physiology. Leukocytes can initiate an inflammatory response in the vascular wall resulting in the release of inflammatory mediators and oxidants (Szekanecz and Koch, 2004; Langer and Chavakis, 2009), which may lead to dysfunction at the blood-endothelium interface. Endothelial dysfunction is an early event in the pathophysiological sequelae of diseases including atherosclerosis, ischemia/reperfusion injury in the heart, and in sepsis multi-organ failure (Ijzerman et al., 2003; Crea et al., 2014; De Backer et al., 2014). Once activated the endothelium releases many mediators that can contribute to leukocyte adherence, coagulation, and changes in barrier function (Ait-Oufella et al., 2010). Through this escalating inflammation, leukocyte-produced reactive oxygen species and released proteolytic enzymes propagate tissue damage and are involved in organ failure (Rossaint and Zarbock, 2015; Santos et al., 2016).

Myeloperoxidase is the most abundant protein in the neutrophil and is a key antimicrobial enzyme released by activated neutrophils (Ysebaert et al., 2000; Klebanoff et al., 2013). During neutrophil activation sequential NADPH oxidase production of hydrogen peroxide and MPO conversion of hydrogen peroxide and chloride anion lead to the production of HOCl. HOCl is a potent antimicrobial agent capable of oxidizing proteins, free amino acids, carbohydrates, DNA, and lipids (Harrison and Schultz, 1976; Hazen et al., 1996; Pattison and Davies, 2006; Pattison et al., 2009). Plasmalogens, a major phospholipid in many human organs, are targeted by HOCl leading to the production of 2-ClFALDs (Dorman et al., 1976; Chilton and Connell, 1988; Ford and Gross, 1989; Murphy et al., 1992; Hazen et al., 1993; Portilla and Creer, 1995; McHowat et al., 1997; Albert et al., 2001; Hsu et al., 2003). 2-ClFALDs can enter the fatty acid-fatty alcohol cycle as an intermediate leading to their oxidation to 2-chlorofatty acids (2-ClFAs) (Rizzo et al., 1987; Wildsmith et al., 2006; Anbukumar et al., 2010; Brahmbhatt et al., 2010). During inflammation, 2-ClFALDs and 2-ClFAs are produced at the site of neutrophil infiltration and likely alter nearby cell function (Ford, 2010).

2-Chlorofatty aldehyde and 2-ClFA have been linked to inflammatory diseases, including endotoxemia and atherosclerosis (Thukkani et al., 2003, 2005; Brahmbhatt et al., 2010; Ford et al., 2016). Activated neutrophils and monocytes isolated from human blood have elevated levels of 2-ClFALD, approaching 20–90 μM (compared to undetectable levels in inactivated neutrophils and monocytes), which represents an approximation of the maximal concentrations at the site of production near the leukocyte-endothelial interface (Thukkani et al., 2002; Anbukumar et al., 2010). Other studies have also shown neutrophil-derived HOCl can target endothelial plasmalogens resulting in endothelial 2-ClFALD production (Thukkani et al., 2002). Chlorinated lipids have been shown to elicit recruitment of macrophages to the lung, disrupt the blood-brain barrier, and induce apoptosis (Wang et al., 2014; Ford et al., 2016; Nusshold et al., 2016). Recently 2-ClFALD was shown to increase leukocyte rolling and adhesion in the mesenteric microcirculation (Yu et al., 2018). Despite these insights into the biological role of chlorinated lipids the role of 2-ClFALD and 2-ClFA elicited endothelial dysfunction remain to be further characterized. In particular, the demonstration that 2-ClFA associates with ARDS in humans with sepsis (Meyer et al., 2017) suggests understanding the role of 2-ClFALD, the precursor of 2-ClFA, on tissue specific endothelium is needed. Accordingly, the present studies were designed to elucidate mechanisms responsible for 2-ClFALD mediated endothelial activation and distinguish mechanisms in endothelial cells from several vascular beds. Furthermore, a novel click chemistry approach was used to determine the subcellular localization of 2-ClFALD in vascular endothelial cells.

## Materials and Methods

### Materials

Cell culture supplies were purchased from Sigma-Aldrich (Vienna, Austria), Lonza (Basel, Switzerland), or Cell Applications Inc. (San Diego, CA, United States). Click-It Cell Reaction Buffer Kit was purchased from Thermo Fisher Scientific (Waltham, MA, United States; catalog no. C10269). Rabbit polyclonal anti-calnexin (catalog no. ab22595), rabbit monoclonal anti- voltage-dependent anion-selective channel 1/porin antibody (anti-VDAC1; catalog no. ab154856), mouse polyclonal anti-golgi matrix protein 130 (anti-GM130; catalog no. ab169276), mouse monoclonal anti-Von Willebrand Factor (anti-VWF; catalog no. ab20435), goat anti-rabbit IgG H&L (Alexa Fluor^®^ 488) (catalog no: 150077), and goat anti-mouse IgG H&L (Alexa Fluor^®^ 488) (catalog no. ab150113) antibodies were purchased from Abcam (Cambridge, United Kingdom). Antibodies to adhesion molecules (mouse monoclonal anti-P-selectin, sc-18834; mouse monoclonal anti-E-selectin, sc-5262; mouse monoclonal anti-intercellular adhesion molecule-1, ICAM-1, sc-53336; mouse monoclonal anti-vascular cell adhesion molecule-1, VCAM-1, sc-13160) were purchased from Santa Cruz Biotechnology (Dallas, TX, United States). All other chemicals were purchased from Sigma-Aldrich or Thermo Fisher Scientific.

### Synthesis of 2-Chlorohexadec-15-ynal (2-ClHDyA)

The alkyne analog of 2-ClHDA (2-ClHDyA), was synthesized according to protocols described previously (Halland et al., 2004; Nusshold et al., 2016). Sequentially, (1) hexadec-7-ynol (Alfa Aesar, cat. B22113) was converted to hexadec-15-ynol using sodium hydride (Sigma-Aldrich, cat. 452912) and diaminopropane (Sigma-Aldrich, cat. D23602) (Nusshold et al., 2016), hexadec-15-ynol was oxidized to HDyA in a solution of 2-iodoxybenzoic acid (Sigma-Aldrich, cat. 661384) in DMSO (Sigma-Aldrich, cat. D2650) (Nusshold et al., 2016), and HDyA was chlorinated using *N-*chlorosuccinimide (Sigma-Aldrich, cat. 109681) and proline (Sigma-Aldrich, cat. P0380) for 16 h (Halland et al., 2004). Products were purified by flash chromatography (30 g silica gel, high purity grade, pore size 60 Å, Sigma-Aldrich, cat. 227196) (Hartman et al., 2018). 2-ClHDyA was subsequently quantitated by GC-FID following conversion to its dimethyl acetal derivative using heptadecanoic acid and its methyl ester derivative as internal standard (Gross, 1985; Albert et al., 2001).

### Endothelial Cell Culture

Human coronary artery endothelial cells (HCAEC, Lonza, cat. CC-2585) and human lung microvascular endothelial cells (HLMVEC, Lonza, cat. CC-2527) were grown in EGM-2MV medium (Lonza, cat. CC-3202). Human renal glomerular endothelial cells (HKEC, Sciencell, cat. 4000) were grown in endothelial cell medium containing 5% FBS (Sciencell, cat. 1001). Endothelial cells were used in experiments at passage 4–8. Cells were treated with lipids in DMSO.

### Localization of 2-ClHDyA in HCAEC and HLMVEC

Cells were grown to confluence on sterile coverslips and treated with 10 μM 2-ClHDyA, or no lipid, for 30 min or 60 min. Cells were washed with PBS, fixed with formalin, and permeabilized with 0.25% Triton X-100 for 10 min. In the case of VDAC1, cells were permeabilized with ice cold 100% methanol for 10 min at −20°C. Cells were subsequently washed with 2% (w/v) BSA in PBS and labeled with 5 μM azide-carboxytetramethylrhodamine (azide-TAMRA) (Sigma-Aldrich; catalog no. 760757) by using the Click-It Cell Reaction Buffer Kit (Thermo Fisher; catalog no. C10269) following manufacturer’s protocols. After click reaction, cells were washed with 2% BSA in PBS. To identify subcellular localization of 2-ClHDyA, cells were incubated with primary antibodies against VDAC1 (1:1000), GM130 (1:142), calnexin (1:1000), and VWF (1:100) overnight at 4°C. Cells were washed three times with PBS for 5 min and incubated with goat anti-mouse IgG secondary antibody (1:500) labeled with Alexa 488 or goat anti rabbit IgG secondary antibody (1:500) for 1 h. The coverslips were mounted onto microscope slides with a Vectashield solution containing 4′,6-diamidino-2-phenylindole (DAPI; Vector Laboratories; catalog no. H1200).

### Confocal Microscopy

A Leica SP5 confocal microscope (Leica Microsystems, Mannheim, Germany) with a 63 × 1.4 oil immersion objective was used to acquire images. Alexa 488 was excited at 488 nm and detected between 500 and 540 nm. Azide-TAMRA fluorescence was excited at 543 nm and detected between 570 and 650 nm. DAPI fluorescence was detected between 440 and 470 nm. Alexa 488 and TAMRA fluorescence signals were acquired simultaneously.

### Adhesion Molecule Surface Expression

Human coronary artery endothelial cells, HLMVEC, and HKEC were grown to confluence and treated with either 10 μM 2-ClHDA or HDA for 30 min (P-selectin), 1 h (E-selectin) or 4 h (ICAM-1 and VCAM-1). Cells were not permeabilized, but were fixed with 1% paraformaldehyde overnight and cell surface expression of adhesion molecules measured as described previously (Meyer et al., 2017; Hartman et al., 2018).

### Platelet and Neutrophil Adherence to Endothelial Cells

Adherence of platelets to either HCAEC, HLMVEC or HKEC was determined as previously described (Verheul et al., 2000). Platelets were isolated from the whole blood of healthy volunteers as previously described (Beckett et al., 2007), and as authorized by Saint Louis University Institutional Review Board Protocol 12369. Platelets were stained with Calcein-AM (2.5 μmol/L; Thermo Fisher, cat. C3100MP) for 15 min at 37°C in the dark. Fluorescence-labeled platelets (50 × 10^6^ cells in 500 μL) were added to endothelial cells and incubated for 20 min at 37°C. Platelet adherence was determined by fluorescence measurement (excitation at 492 nm, emission at 535 nm). Neutrophils were prepared from whole blood of healthy volunteers as previously described (Thukkani et al., 2002; Duerr et al., 2015), and as authorized by Saint Louis University Institutional Review Board Protocol 10014. 2 × 10^6^ neutrophils were added to endothelial cells and incubated for 20 min. Neutrophil adherence was measured as MPO activity using 1,9-dimethyl-methylene blue, and absorbance measured at 460 nm as described previously (Meyer et al., 2017; Hartman et al., 2018).

### Endothelial Cell Permeability

Endothelial cells were grown to confluence on Transwell polycarbonate filters (Corning Inc., Corning, NY, United States) mounted in a chamber insert. Resistance across cells was monitored daily using an EVOM volt-ohmmeter (World Precision Instruments). The lipids, 2-ClHDA or HDA, (10 μM) were added to each well then the resistance across each well was monitored up to 24 h.

### Cell Viability Assay

The metabolic activity of HLMVEC was examined using the 3-(4,5-dimethyl-2-thiazolyl)-2,5-diphenyl-2H-tetrazolium bromide (MTT) assay as previously described (Hartman et al., 2018) to test changes in metabolic activity elicited by either no lipid, 2-ClHDA or 2-ClHDyA (10 μM).

### Statistical Analyses

ANOVA with the Tukey’s *post hoc* test was used for comparisons between three groups. All data are presented as mean ± SEM unless otherwise noted.

## Results

### Effect of 2-ClHDA on Surface Expression of Adhesion Molecules as Well as Neutrophil and Platelet Adherence

**FIGURE 1:**
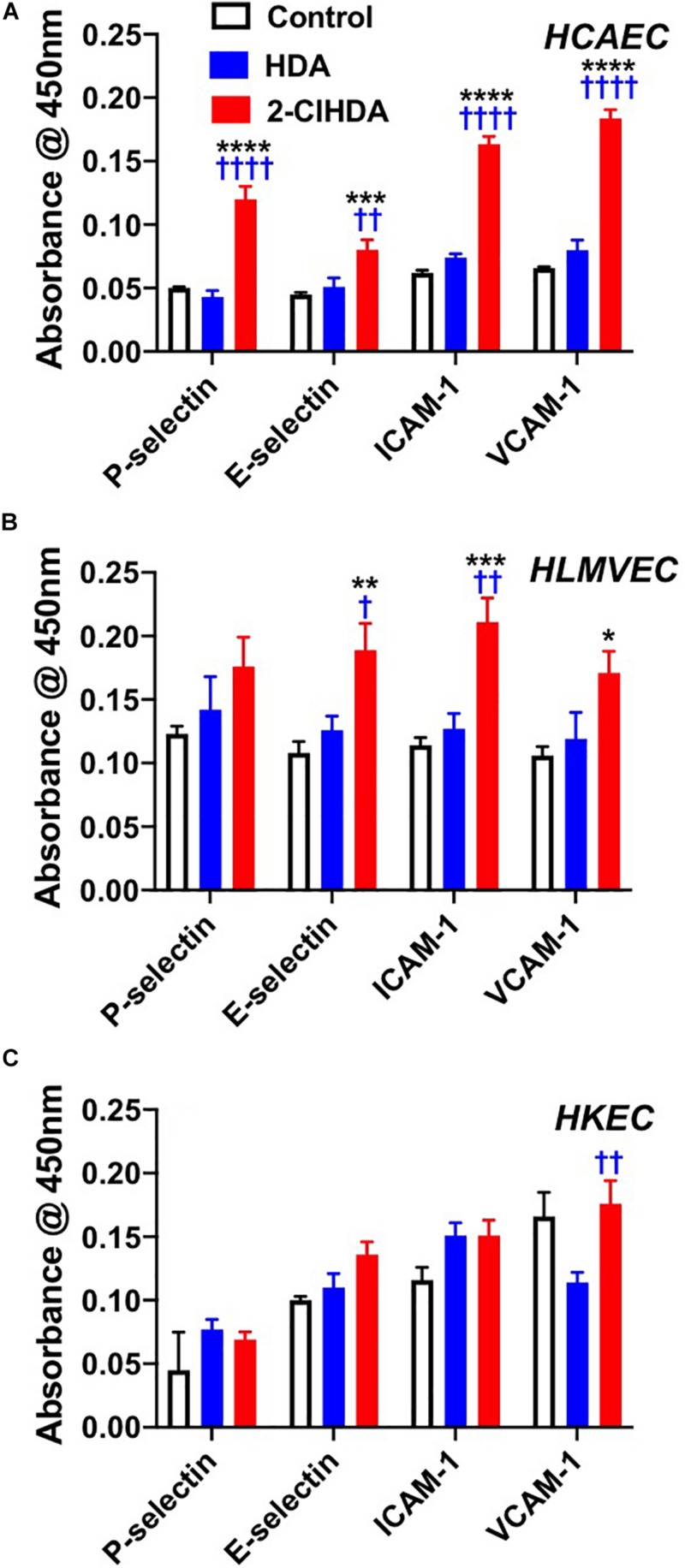
Effects of 2-ClHDA on selectin, ICAM-1 and VCAM-1 surface expression in HCAEC **(A)**, HLMVEC **(B)**, and HKEC **(C)**. Cells were treated with 10 μM HDA, 10 μM 2-ClHDA or vehicle for 0.5 (P-selectin), 1 h (E-selectin) or 4 h (VCAM-1 and ICAM-1). Cells were incubated with primary antibodies and then incubated with HRP-conjugated secondary antibodies. Surface expression of selectins, ICAM-1 and VCAM-1 was measured spectrophotometrically after addition of TMB substrate. Values are normalized to secondary antibody alone. *n* = 4 for each treatment, mean ± SEM. *P < 0.05, **P < 0.01, ***P < 0.001, and ****P < 0.0001 for comparisons with control treatment. ^†^ *P* < 0.05, ^††^ *P* < 0.01, and ^††††^ *P* < 0.0001 for comparisons with HDA treatment.

Previous studies have shown 2-ClHDA increases leukocyte rolling and adhesion in the mesenteric microcirculation (Yu et al., 2018). To better understand mechanisms responsible for *in vivo* leukocyte rolling and adhesion we examined the role of 2-ClHDA as a mediator of both adhesion molecule surface expression as well as adhesion of neutrophils and platelets in isolated endothelial cells. Furthermore these studies were performed with human endothelial cells from several vascular beds including HCAEC, HLMVEC and HKEC. Data shown in Figure 1A highlight the responsiveness of HCAEC to 2-ClHDA treatment. Significant increases in surface expression of P-selectin, E-selectin, ICAM-1, and VCAM-1 in response to 2-ClHDA compared to treatments with either vehicle (control) or HDA. The differential response to 2-ClHDA compared to HDA reveals the importance of the α-carbon chlorination of the aldehyde. In comparison to HCAEC, HLMVEC (Figure 1B) had a weaker response to 2-ClHDA treatment, but did show significant increases in E-selectin, ICAM-1, and VCAM-1 when compared to control conditions. Again, HDA did not cause surface expression of these molecules in HLMVEC. In a separate study, cell surface expression of adhesion molecules in response to 2-ClHDA was compared to LPS incubation (50 ng/ml) in HLMVEC. Similar, but smaller, increases were observed in response to 2-ClHDA when compared to LPS for P-selectin (0.170 ± 0.005 2-ClHDA vs. 0.188 ± 0.005 LPS), E-selectin (0.135 ± 0.003 2-ClHDA vs. 0.258 ± 0.008 LPS), ICAM-1 (0.189 ± 0.006 2-ClHDA vs. 0.258 ± 0.006 LPS), and VCAM-1 (0.219 ± 0.007 2-ClHDA vs. 0.253 ± 0.006 LPS). Surface expression of the selectins and ICAM-1 did not significantly increase in HKEC in comparison to control conditions (Figure 1C).

**FIGURE 2:**
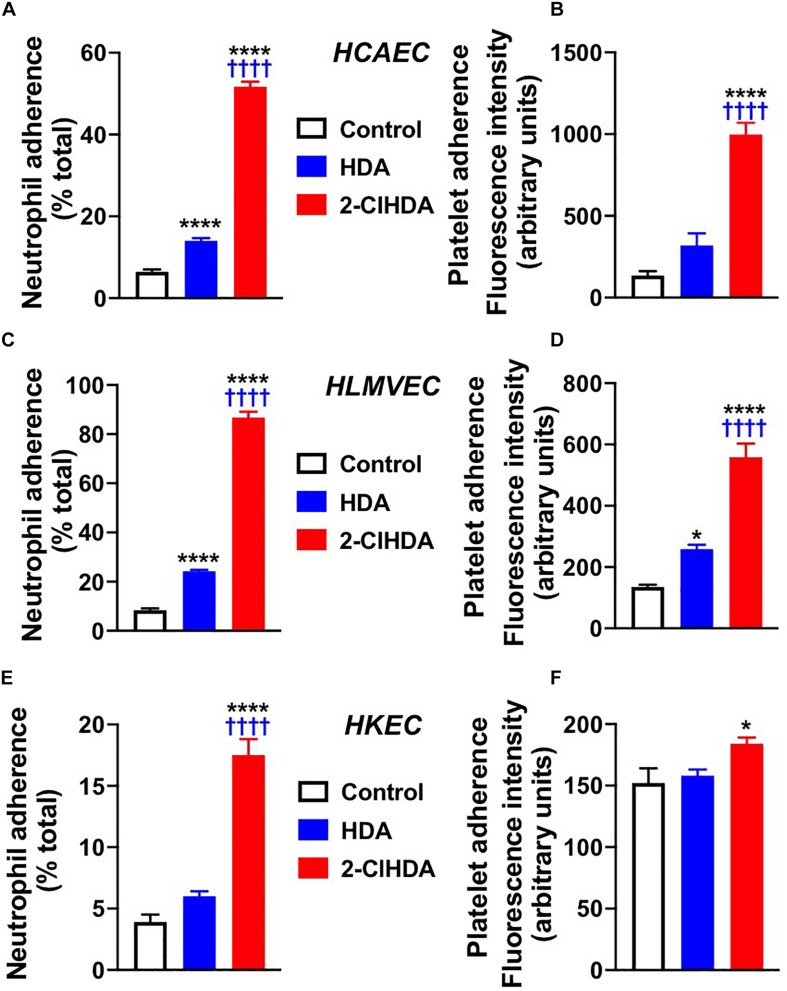
Effects of 2-ClHDA on Neutrophil **(A,C,E)** and Platelet **(B,D,F)** Adherence to HCAEC **(A,B)**, HLMVEC **(C,D)** and HKEC **(E,F)**. Cells were treated with 10 μM HDA, 10 μM 2-ClHDA or vehicle for 4 h. Subsequently endothelial cells were incubated with either freshly isolated human neutrophils or platelets for 20 min to assess adherence. *n* = 4 for each treatment, mean ± SEM. * *P* < 0.05, and **** *P* < 0.0001 for comparisons with control treatment. ^††††^ *P* < 0.0001 for comparisons with HDA treatment.

Selectin adhesion molecules mediate the adherence of leukocytes to the endothelium (Etzioni, 1996; Peyvandi et al., 2011). Because these molecules are increased following 2-ClHDA treatment, the adherence of neutrophils and platelets to endothelium after 2-ClHDA treatment was examined. In concert with elevated surface expression of adhesion molecules, both neutrophil and platelet adhesion to HCAEC and HLMVEC in response to 2-ClHDA was robustly increased in both HCAEC and HLMVEC in comparison to treatments with vehicle as well as in response to HDA (Figures 2A–D). Interestingly although 2-ClHDA mediated neutrophil adherence to HCAEC and HLMVEC was ∼4-fold greater than that elicited by HDA, significant adherence of neutrophils in response to HDA was detected in comparison to vehicle treatments. Although both platelet and neutrophil adherence to HKEC was significantly increased in response to 2-ClHDA treatments (Figures 2E,F), the response in HKEC was weaker compared to that observed in HCAEC and HLMVEC. Additionally HDA did not elicit neutrophil or platelet adhesion to HKEC.

### Endothelial Cell Monolayer Permeability in Response to 2-ClHDA

**FIGURE 3:**
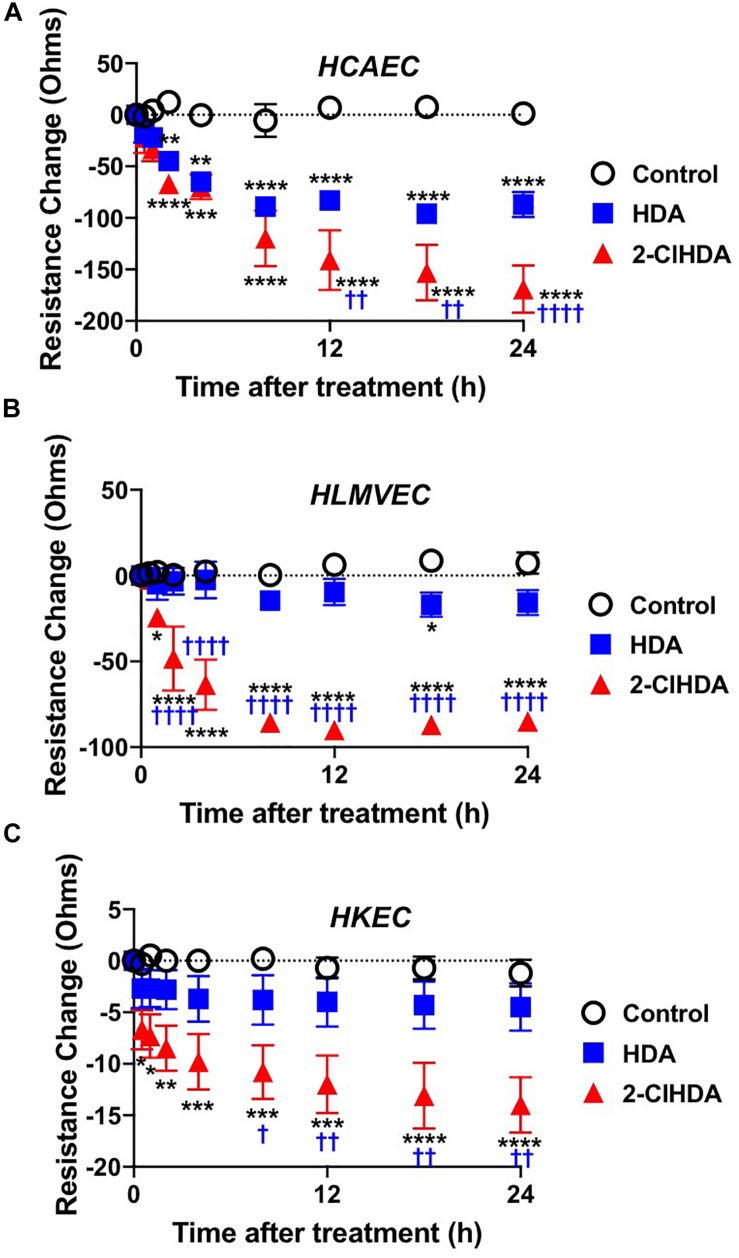
Permeability Barrier of HCAEC **(A)**, HLMVEC **(B)**, and HKEC **(C)**. Cells were grown to confluence on Transwell polycarbonate filters mounted in a chamber insert. Resistance across the cells was monitored daily using an EVOM volt-ohmmeter. Once the resistance remained constant for 3 consecutive days, experiments were performed. 10 μM lipids were added to each well then the resistance across each well was monitored at 0.5, 1, 2, 4, 8, 12, 18, and 24 h. *n* = 6 for each treatment, mean ± SEM. * *P* < 0.05, ** *P* < 0.01, *** *P* < 0.001, **** *P* < 0.0001 for comparisons with control treatment. ^†^ *P* < 0.05, ^††^ *P* < 0.01 and ^††††^ *P* < 0.0001 for comparisons with HDA treatment.

Similar to conditions comparing adhesion molecule surface expression and neutrophil adhesion to endothelial cells, the impact of 2-ClHDA on endothelial permeability barrier function was determined. HCAEC, HLMVEC, and HKEC all significantly lost barrier function, measured as changes in electrical resistance, in response to 2-ClHDA treatments in comparison to vehicle control treatment (Figures 3A–C). Comparing the response of the three endothelial cell lines to 2-ClHDA indicated HCAEC > HLMVEC > HKEC. Additionally, HCAEC was uniquely susceptible to HDA-mediated loss of barrier function albeit this loss was not as great as that elicited by 2-ClHDA (Figure 3A). Changes in electrical resistance in response to 2-ClHDA were similar to those observed when HLMVEC were incubated with LPS (50 ng/ml) for up to 24 h (data not shown).

### Subcellular Localization of 2-ClHDyA

**FIGURE 4:**
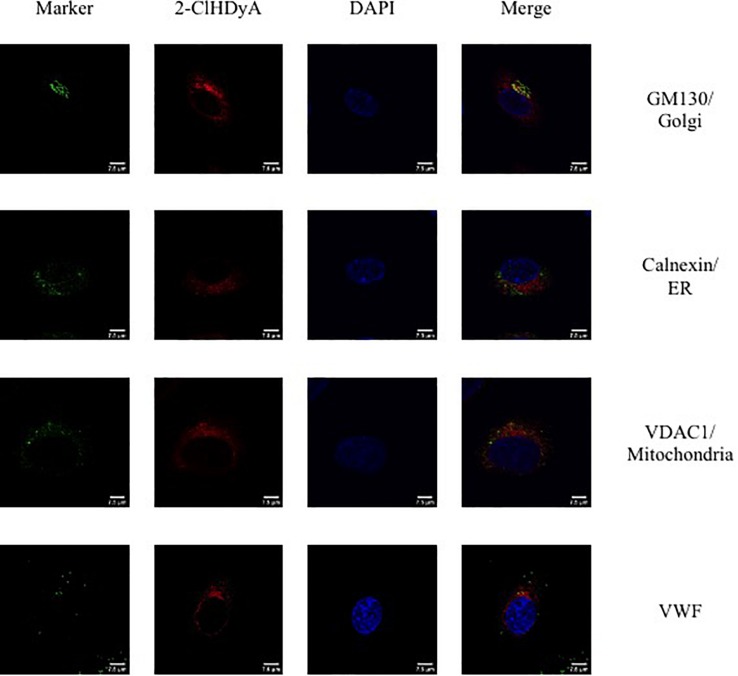
Subcellular localization of 2-ClHDyA in HLMVEC at 30 min. Cells were grown to confluence on sterile coverslips and treated with 10 μM 2-ClHDyA for 30 min. Cells were fixed with formalin, permeabilized with Triton X-100, except for VDAC1 when cells were permeabilized with ice cold 100% methanol and clicked with azide TAMRA (red). Cells were incubated with primary antibodies against GM130 (Golgi), VWF, calnexin (ER), and VDAC1 (mitochondria), then labeled with Alexa 488 labeled secondary antibodies (green). Cells were mounted in DAPI-containing solution (blue) and imaged with a Leica SP5 microscope. All fluorescence was taken simultaneously.

**FIGURE 5:**
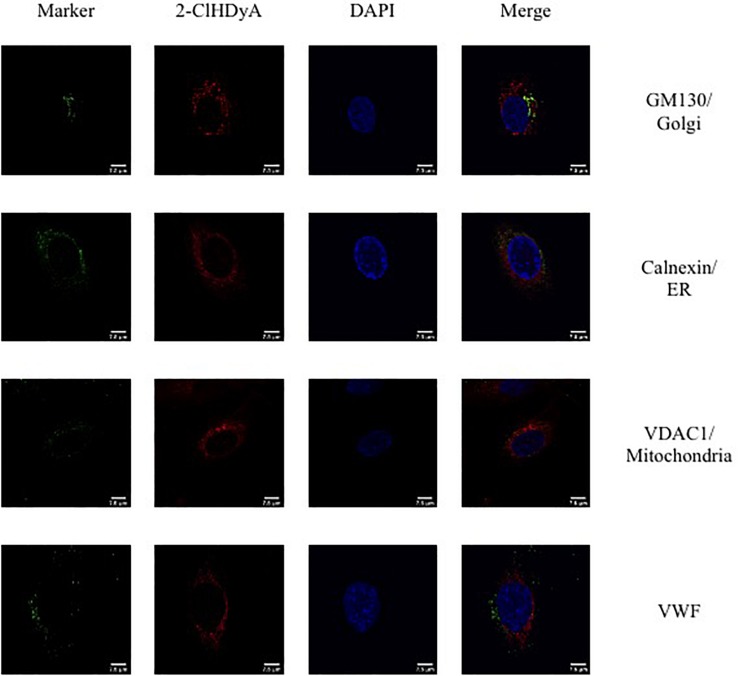
Subcellular localization of 2-ClHDyA in HLMVEC at 60 min. Cells were grown to confluence on sterile coverslips and treated with 10 μM 2-ClHDyA for 60 min. Cells were fixed with formalin, permeabilized with Triton X-100, except for VDAC1 when cells were permeabilized with ice cold 100% methanol and clicked with azide TAMRA (red). Cells were incubated with primary antibodies against GM130 (Golgi), VWF, calnexin (ER), and VDAC1 (mitochondria), then labeled with Alexa 488 labeled secondary antibodies (green). Cells were mounted in DAPI-containing solution (blue) and imaged with a Leica SP5 microscope. All fluorescence was taken simultaneously.

**FIGURE 6:**
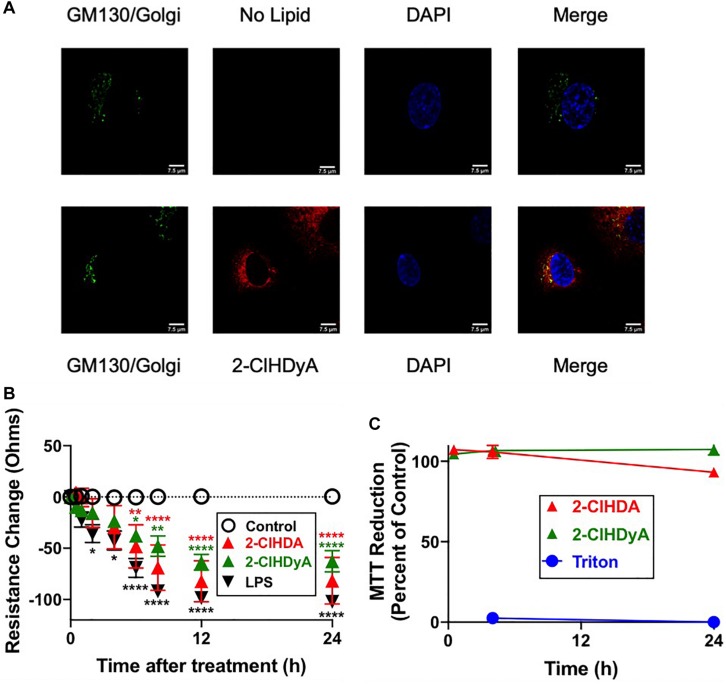
2-ClHDyA in HLMVEC: Specificity of TAMRA-Azide Click Reaction and Comparisons to 2-ClHDA Changes in Barrier Function and MTT Activity. **(A)** Cells were grown to confluence on sterile coverslips and treated with 10 μM 2-ClHDyA (bottom panel) or no lipid (top panel) for 60 min. Indicated GM130 (Golgi) staining and DAPI staining as well as confocal microscopy were performed as described in Figure 5. **(B)** Cells were grown to confluence on Transwell polycarbonate filters mounted in a chamber insert. Resistance across the cells was monitored daily using an EVOM volt-ohmmeter. Once the resistance remained constant for 3 consecutive days, experiments were performed. Either 10 μM lipids or LPS (50 ng/ml) were added to each well then the resistance across each well was monitored for indicated times. *n* = 6 for each treatment, mean ± SEM. In some cases the SEM is within the symbol. * *P* < 0.05, ** *P* < 0.01, **** *P* < 0.0001 for comparisons with control treatment for each of the lipids or LPS as indicated by color of symbols. **(C)** HLMVEC cells were treated with either 2-ClHDA or 2-ClHDyA (10 μM) for indicated time point at 37°C. Metabolic activity of HLMVEC was measured using an MTT assay following manufacturer’s protocol. MTT reduction is expressed as percent of control MTT reduction (vehicle only, designated as 100%). *n* = 4 for each treatment, mean ± SEM. SEM is within the symbol of mean for majority of data points.

**FIGURE 7:**
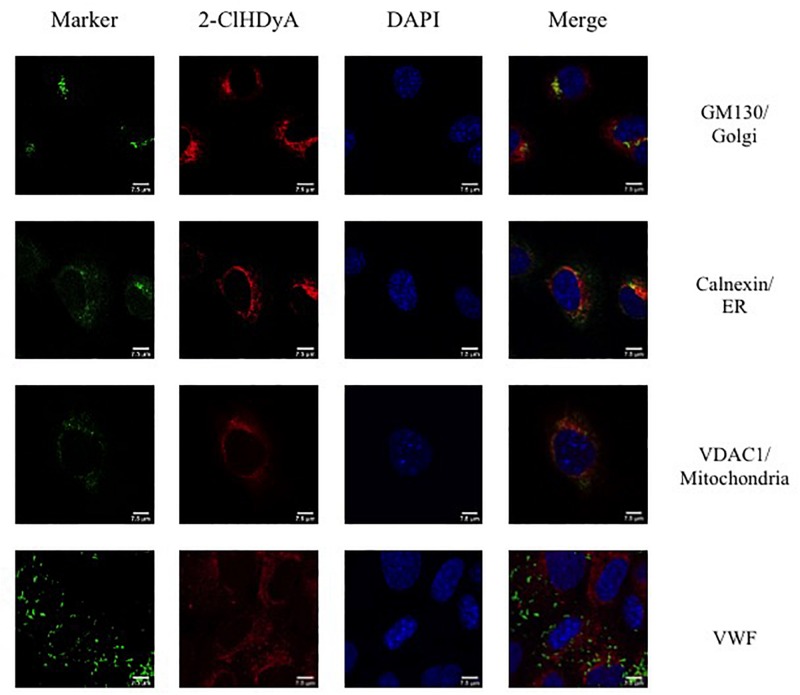
Subcellular localization of 2-ClHDyA in HCAEC at 30 min. Cells were grown to confluence on sterile coverslips and treated with 10 μM 2-ClHDyA for 30 min. Cells were fixed with formalin, permeabilized with Triton X-100, except for VDAC1 when cells were permeabilized with ice cold 100% methanol and clicked with azide TAMRA (red). Cells were incubated with primary antibodies against GM130 (Golgi), VWF, calnexin (ER), and VDAC1 (mitochondria), then labeled with Alexa 488 labeled secondary antibodies (green). Cells were mounted in DAPI-containing solution (blue) and imaged with a Leica SP5 microscope. All fluorescence was taken simultaneously.

**FIGURE 8:**
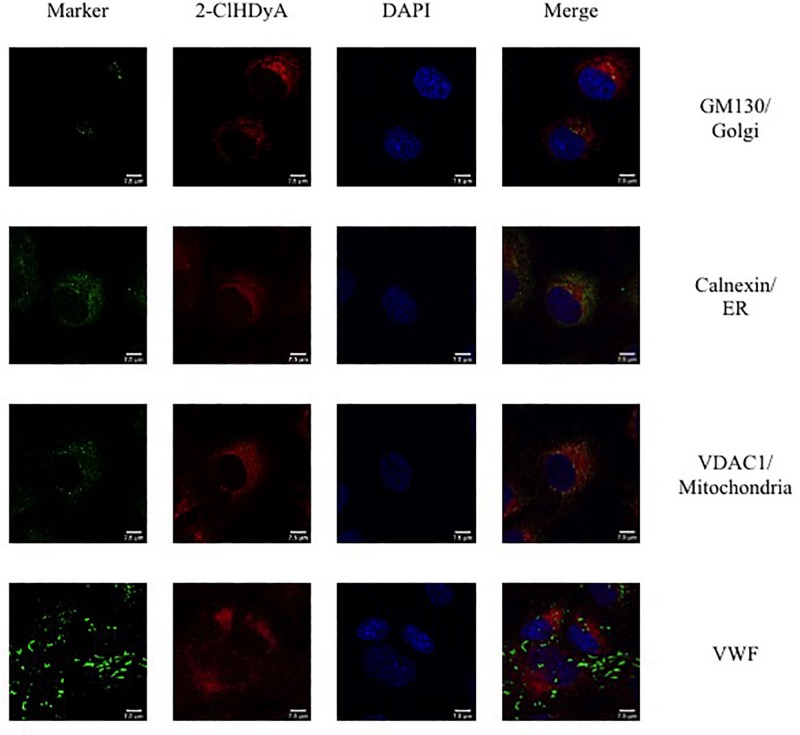
Subcellular localization of 2-ClHDyA in HCAEC at 60 min. Cells were grown to confluence on sterile coverslips and treated with 10 μM 2-ClHDyA for 60 min. Cells were fixed with formalin, permeabilized with Triton X-100, except for VDAC1 when cells were permeabilized with ice cold 100% methanol and clicked with azide TAMRA (red). Cells were incubated with primary antibodies against GM130 (Golgi), VWF, calnexin (ER), and VDAC1 (mitochondria), then labeled with Alexa 488 labeled secondary antibodies (green). Cells were mounted in DAPI-containing solution (blue) and imaged with a Leica SP5 microscope. All fluorescence was taken simultaneously.

Since HLMVEC and HCAEC gave robust responses to 2-ClHDA, additional studies were performed to examine the subcellular distribution of 2-ClHDA in these cells. For these studies the click-chemistry analog of 2-ClHDA, 2-ClHDyA, was used. Following either a 30 min or 60 min incubation of 2-ClHDyA with endothelial cells, cells were fixed and then click reactions were performed using an azide with TAMRA reporter. Colocalization analyses with known organelle markers showed that 2-ClHDyA localized with the ER, Golgi and mitochondria as indicated by calnexin, GM130 and VDAC1 at both 30 and 60 min in HLMVEC (Figures 4, 5). Additional control experiments showed azide-TAMRA specifically reacts with HLMVEC treated with 2-ClHDyA (Figure 6A, lower panel) and was undetectable in cells that were not treated with 2-ClHDyA (Figure 6A, upper panel). Additional test also showed 2-ClHDyA and 2-ClHDA both elicited barrier dysfunction similar to LPS in HLMVEC (Figure 6B) and did not alter cell metabolic activity as determined by MTT assay (Figure 6C). A similar distribution of 2-ClHDyA with ER, Golgi and mitochondria were observed in HCAEC (Figures 7, 8). 2-ClHDyA did not colocalize with the Weibel-Palade bodies (detected with VWF in HCAEC) or VWF storage granules in HLMVEC.

## Discussion

Much of the biological role of chlorinated lipids on endothelial activation have focused on the longer lived chlorinated lipid, 2-ClFA, while the mechanisms underlying biological effects of the 2-ClFA precursor, 2-ClFALD, remain to be explored. Using intravital microscopy in the rat we recently showed 2-ClFALD elicits leukocyte rolling and adhesion, as well as permeability changes, in the mesenteric microcirculation. Others have also shown 2-ClFALD causes blood brain barrier dysfunction, but did not examine leukocyte adhesion (Nusshold et al., 2016). These studies also showed 2-ClFALD associated with Golgi, ER and mitochondria in an immortalized human brain endothelial cell line. To enhance our understanding of the biological role of 2-ClFALD we now show 2-ClFALD elicits endothelial activation in primary human endothelial cells from three disparate vascular beds (i.e., coronary artery, lung microvascular, and kidney) resulting in adhesion molecule surface expression, neutrophil and platelet adherence, and permeability barrier dysfunction. These findings are significant since they provide important information on tissue beds associated with multi-organ failure during sepsis, and in particular plasma 2-ClFA levels associate with ARDS and 30-day mortality in human sepsis (Meyer et al., 2017).

Human coronary artery endothelial cells were very sensitive to activation by the 2-ClFALD, 2-ClHDA at a concentration (10 μM), which is below maximal concentrations estimated at the leukocyte-endothelial interface (20–90 μM) (Thukkani et al., 2002; Anbukumar et al., 2010). Robust surface expression of P-selectin, E-selectin, ICAM-1, and VCAM-1 were observed. Neutrophil and platelet adhesion to HCAEC accompanied the surface expression of adhesion molecules in response to 2-ClHDA. Interestingly there was robust adhesion of neutrophils and platelets to 2-ClHDA treated HLMVEC and HKEC in the absence of robust increases of surface expression of adhesion molecules. Disparate responses to agonists by endothelial cells originating from different vascular beds have previously been observed (Scott and Patel, 2013), and thus these results most likely represent differences in selectin density and mobilization in response to 2-ClHDA in these cell systems. It can also be speculated that at least for neutrophils, adherence may involve neutrophil netosis, which has been shown to be accelerated by the 2-ClHDA oxidation product, 2-chlorofatty acid (Palladino et al., 2018). Further investigations need to determine if there are novel protein ligands and mechanisms that may be responsible for neutrophils and platelet adherence to 2-ClHDA treated endothelial cells.

We previously used the click-chemistry analog of 2-ClFA to show it selectively associates with the Weibel-Palade bodies of HCAEC and subsequently elicits the cascade of events including P-selectin surface expression and neutrophil adhesion (Hartman et al., 2018). The association of 2-ClFA to the Weibel-Palade body suggested the localization may have a key role in mobilizing these storage granules with subsequent surface expression of P-selectin. Similar to 2-ClFA, 2-ClHDA also caused P-selectin surface expression and neutrophil adhesion. However, unlike the click analog of 2-ClFA, 2-ClHDyA did not localize to the HCAEC Weibel-Palade bodies. 2-ClHDyA localized to the ER, Golgi and mitochondria of both HCAEC and HLMVEC. Thus the mechanism responsible for 2-ClHDA mediated mobilization of Weibel-Palade bodies and other granules containing adherence molecules remain to be resolved. In this respect it also should be noted that the HLMVEC contain P-selectin and VWF in separate storage granules (Ochoa et al., 2010; Wu et al., 2014). Future studies need to be performed to determine the mechanism responsible for 2-ClHDA as well as 2-ClFA elicited endothelial activation. These studies using confocal microscopy highlight that there may be unique mechanisms responsible for 2-ClHDA and 2-ClFA mediated endothelial activation. Additionally the relationship of chlorinated lipids and their association with adhesion molecule receptors needs to be examined.

2-Chlorohexadecanal caused permeability barrier dysfunction in HCAEC, HLMVEC, and HKEC. HDA did have some modest effects in causing endothelial permeability barrier dysfunction, but not to the degree observed with 2-ClHDA treatment. The mechanism responsible for permeability barrier dysfunction may involve adheren dysfunction with intercellular gap formation.

These studies demonstrate that the initial product of HOCl targeting plasmalogens, 2-ClFALD, activates endothelial cells resulting in adhesion molecule surface expression, neutrophil and platelet adherence and loss of permeability barrier function. There are modest differences in the responses to 2-ClFALD by endothelial cells from three origins including human coronary arteries, human lung, and human kidney. In conjunction with our previous studies using intravital microscopy (Yu et al., 2018), these data suggest this is a key mechanism through which 2-ClFALD can elicit *in vivo* endothelial dysfunction leading to microcirculatory collapse. Several key observations were also made indicating there are additional key gaps that need to be examined in the future including the precise mechanism that 2-ClFALD elicits adhesion molecule surface expression and the mechanisms responsible for platelet and neutrophil adherence to 2-ClFALD treated endothelial cells in the absence of a response with adhesion molecule surface expression (e.g., in HKEC).

## Data Availability Statement

All datasets generated for this study are included in the article/supplementary material.

## Ethics Statement

The studies involving human participants were reviewed and approved by the Saint Louis University IRB. The patients/participants provided their written informed consent to participate in this study.

## Author Contributions

DF was responsible for oversight of all aspects of studies and manuscript preparation. JM performed endothelial cell functional assays and manuscript preparation. SS performed endothelial cell sub cellular localization studies and manuscript preparation.

## Conflict of Interest

The authors declare that the research was conducted in the absence of any commercial or financial relationships that could be construed as a potential conflict of interest.

## Abbreviations

2-ClFALD: 2-chlorofatty aldehyde
2-ClHDA: 2-chlorohexadecanal
2-ClHDyA: 2-chlorohexadec-15-ynal
HCAEC: human coronary artery endothelial cell
HDA: hexadecanal
HDyA: hexadec-15-ynal
MPO: myeloperoxidase
PFB-Br: pentafluorobenzyl bromide
VWF: von Willebrand factor.

## References

[B1] Ait-Oufella H. Maury E. Lehoux S. Guidet B. Offenstadt G. (2010). The endothelium: physiological functions and role in microcirculatory failure during severe sepsis. *Intensive Care Med.* 36 1286–1298. 10.1007/s00134-010-1893-6 20443110

[B2] Albert C. J. Crowley J. R. Hsu F. F. Thukkani A. K. Ford D. A. (2001). Reactive chlorinating species produced by myeloperoxidase target the vinyl ether bond of plasmalogens: identification of 2-chlorohexadecanal. *J. Biol. Chem.* 276 23733–23741. 10.1074/jbc.M101447200 11301330

[B3] Anbukumar D. S. Shornick L. P. Albert C. J. Steward M. M. Zoeller R. A. Neumann W. L. (2010). Chlorinated lipid species in activated human neutrophils: lipid metabolites of 2-chlorohexadecanal. *J. Lipid Res.* 51 1085–1092. 10.1194/jlr.M003673 20019386 PMC2853435

[B4] Beckett C. S. Kell P. J. Creer M. H. McHowat J. (2007). Phospholipase a2-catalyzed hydrolysis of plasmalogen phospholipids in thrombin-stimulated human platelets. *Thromb. Res.* 120 259–268. 10.1016/j.thromres.2006.09.005 17055038 PMC2204082

[B5] Brahmbhatt V. V. Albert C. J. Anbukumar D. S. Cunningham B. A. Neumann W. L. Ford D. A. (2010). {omega}-oxidation of {alpha}-chlorinated fatty acids: Identification of {alpha}-chlorinated dicarboxylic acids. *J. Biol. Chem.* 285 41255–41269. 10.1074/jbc.M110.147157 20956542 PMC3009851

[B6] Chilton F. H. Connell T. R. (1988). 1-ether-linked phosphoglycerides. Major endogenous sources of arachidonate in the human neutrophil. *J. Biol. Chem.* 263 5260–5265. 3128538

[B7] Crea F. Camici P. G. Bairey Merz C. N. (2014). Coronary microvascular dysfunction: an update. *Eur. Heart J.* 35 1101–1111. 24366916 10.1093/eurheartj/eht513 PMC4006091

[B8] De Backer D. Orbegozo Cortes D. Donadello K. Vincent J.-L. (2014). Pathophysiology of microcirculatory dysfunction and the pathogenesis of septic shock. *Virulence* 5 73–79. 10.4161/viru.26482 24067428 PMC3916386

[B9] Dorman R. V. Dreyfus H. Freysz L. Horrocks L. A. (1976). Ether lipid content of phosphoglycerides from the retina and brain of chicken and calf. *Biochim. Biophys. Acta* 486 55–59. 10.1016/0005-2760(77)90069-8 1009135

[B10] Duerr M. A. Aurora R. Ford D. A. (2015). Identification of glutathione adducts of alpha-chlorofatty aldehydes produced in activated neutrophils. *J. Lipid Res.* 56 1014–1024. 10.1194/jlr.M058636 25814023 PMC4409278

[B11] Etzioni A. (1996). Adhesion molecules-their role in health and disease. *Pediatr. Res.* 39 191–198. 8825786 10.1203/00006450-199602000-00001

[B12] Ford D. A. (2010). Lipid oxidation by hypochlorous acid: chlorinated lipids in atherosclerosis and myocardial ischemia. *Clin. Lipidol.* 5 835–852. 10.2217/clp.10.68 21339854 PMC3041592

[B13] Ford D. A. Gross R. W. (1989). Plasmenylethanolamine is the major storage depot for arachidonic acid in rabbit vascular smooth muscle and is rapidly hydrolyzed after angiotensin ii stimulation. *Proc. Natl. Acad. Sci. U.S.A.* 86 3479–3483. 10.1073/pnas.86.10.3479 2498871 PMC287161

[B14] Ford D. A. Honavar J. Albert C. J. Duerr M. A. Oh J.-Y. Doran S. (2016). Formation of chlorinated lipids post-chlorine gas exposure. *J. Lipid Res.* 57 1529–1540. 10.1194/jlr.M069005 27324796 PMC4959868

[B15] Gross R. W. (1985). Identification of plasmalogen as the major phospholipid constituent of cardiac sarcoplasmic reticulum. *Biochemistry* 24 1662–1668. 10.1021/bi00328a014 3159423

[B16] Halland N. Braunton A. Bachmann S. Marigo M. Jorgensen K. A. (2004). Direct organocatalytic asymmetric alpha-chlorination of aldehydes. *J. Am. Chem. Soc.* 126 4790–4791. 10.1021/ja049231m 15080678

[B17] Harrison J. E. Schultz J. (1976). Studies on the chlorinating activity of myeloperoxidase. *J. Biol. Chem.* 251 1371–1374. 176150

[B18] Hartman C. L. Duerr M. A. Albert C. J. Neumann W. L. McHowat J. Ford D. A. (2018). 2-chlorofatty acids induce weibel-palade body mobilization. *J. Lipid Res.* 59 113–122. 10.1194/jlr.M080200 29167411 PMC5748502

[B19] Hazen S. L. Hall C. R. Ford D. A. Gross R. W. (1993). Isolation of a human myocardial cytosolic phospholipase a_2_ isoform. Fast atom bombardment mass spectroscopic and reverse-phase high pressure liquid chromatography identification of choline and ethanolamine glycerophospholipid substrates. *J. Clin. Invest.* 91 2513–2522. 10.1172/JCI116487 8514863 PMC443312

[B20] Hazen S. L. Hsu F. F. Duffin K. Heinecke J. W. (1996). Molecular chlorine generated by the myeloperoxidase-hydrogen peroxide-chloride system of phagocytes converts low density lipoprotein cholesterol into a family of chlorinated sterols. *J. Biol. Chem.* 271 23080–23088. 10.1074/jbc.271.38.23080 8798498

[B21] Hsu F. F. Turk J. Thukkani A. K. Messner M. C. Wildsmith K. R. Ford D. A. (2003). Characterization of alkylacyl, alk-1-enylacyl and lyso subclasses of glycerophosphocholine by tandem quadrupole mass spectrometry with electrospray ionization. *J. Mass Spectrom.* 38 752–763. 10.1002/jms.491 12898655

[B22] Ijzerman R. G. Jongh R. T. D. Beijk M. A. M. Van Weissenbruch M. M. Delemarre-van De Waal H. A. Serné E. H. (2003). Individuals at increased coronary heart disease risk are characterized by an impaired microvascular function in skin. *Eur. J. Clin. Invest.* 33 536–542. 10.1046/j.1365-2362.2003.01179.x 12814388

[B23] Klebanoff S. J. Kettle A. J. Rosen H. Winterbourn C. C. Nauseef W. M. (2013). Myeloperoxidase: a front-line defender against phagocytosed microorganisms. *J. Leukocyte Biol.* 93 185–198. 10.1189/jlb.0712349 23066164 PMC3545676

[B24] Langer H. F. Chavakis T. (2009). Leukocyte-endothelial interactions in inflammation. *J. Cell. Mol. Med.* 13 1211–1220. 19538472 10.1111/j.1582-4934.2009.00811.x PMC2861890

[B25] McHowat J. Jones J. H. Creer M. H. (1997). Gradient elution reversed-phase chromatographic isolation of individual glycerophospholipid molecular species. *J. Chromatogr. B Biomed. Sci. Appl.* 702 21–32. 10.1016/s0378-4347(97)00386-1 9449552

[B26] Meyer N. J. Reilly J. P. Feng R. Christie J. D. Hazen S. L. Albert C. J. (2017). Myeloperoxidase-derived 2-chlorofatty acids contribute to human sepsis mortality via acute respiratory distress syndrome. *J. Clin. Invest. Insight* 2:96432 10.1172/jci.insight.96432 PMC5752281 29212955

[B27] Murphy E. J. Joseph L. Stephens R. Horrocks L. A. (1992). Phospholipid composition of cultured human endothelial cells. *Lipids* 27 150–153. 10.1007/bf02535816 1315902

[B28] Nusshold C. Ullen A. Kogelnik N. Bernhart E. Reicher H. Plastira I. (2016). Assessment of electrophile damage in a human brain endothelial cell line utilizing a clickable alkyne analog of 2-chlorohexadecanal. *Free Radic. Biol. Med.* 90 59–74. 10.1016/j.freeradbiomed.2015.11.010 26577177 PMC6392177

[B29] Ochoa C. D. Wu S. Stevens T. (2010). New developments in lung endothelial heterogeneity: von willebrand factor, p-selectin, and the weibel-palade body. *Semin. Thromb. Hemost.* 36 301–308. 10.1055/s-0030-1253452 20490980 PMC2917989

[B30] Palladino E. N. D. Katunga L. A. Kolar G. R. Ford D. A. (2018). 2-chlorofatty acids: lipid mediators of neutrophil extracellular trap formation. *J. Lipid Res.* 59 1424–1432. 10.1194/jlr.M084731 29739865 PMC6071778

[B31] Pattison D. I. Davies M. J. (2006). Reactions of myeloperoxidase-derived oxidants with biological substrates: gaining chemical insight into human inflammatory diseases. *Curr. Med. Chem.* 13 3271–3290. 10.2174/092986706778773095 17168851

[B32] Pattison D. I. Hawkins C. L. Davies M. J. (2009). What are the plasma targets of the oxidant hypochlorous acid? A kinetic modeling approach. *Chem. Res. Toxicol.* 22 807–817. 10.1021/tx800372d 19326902

[B33] Peyvandi F. Garagiola I. Baronciani L. (2011). Role of von willebrand factor in the haemostasis. *Blood Transfusion* 9 s3–s8. 10.1016/j.thromres.2017.07.018 21839029 PMC3159913

[B34] Portilla D. Creer M. H. (1995). Plasmalogen phospholipid hydrolysis during hypoxic injury of rabbit proximal tubules. *Kidney Int.* 47 1087–1094. 10.1038/ki.1995.155 7783405

[B35] Rizzo W. B. Craft D. A. Dammann A. L. Phillips M. W. (1987). Fatty alcohol metabolism in cultured human fibroblasts. Evidence for a fatty alcohol cycle. *J. Biol. Chem.* 262 17412–17419. 3320042

[B36] Rossaint J. Zarbock A. (2015). Pathogenesis of multiple organ failure in sepsis. *Crit. Rev. Immunol.* 35 277–291. 10.1615/critrevimmunol.2015015461 26757392

[B37] Santos S. S. Carmo A. M. Brunialti M. K. Machado F. R. Azevedo L. C. Assuncao M. (2016). Modulation of monocytes in septic patients: preserved phagocytic activity, increased ros and no generation, and decreased production of inflammatory cytokines. *Intensive Care Med.* 4:5 10.1186/s40635-016-0078-1 PMC4754229 26879814

[B38] Scott D. W. Patel R. P. (2013). Endothelial heterogeneity and adhesion molecules n-glycosylation: implications in leukocyte trafficking in inflammation. *Glycobiology* 23 622–633. 10.1093/glycob/cwt014 23445551

[B39] Szekanecz Z. Koch A. E. (2004). Vascular endothelium and immune responses: implications for inflammation and angiogenesis. *Rheum. Dis. Clin. North Am.* 30 97–114. 10.1016/S0889-857X(03)00116-9 15061570

[B40] Thukkani A. K. Albert C. J. Wildsmith K. R. Messner M. C. Martinson B. D. Hsu F. F. (2003). Myeloperoxidase-derived reactive chlorinating species from human monocytes target plasmalogens in low density lipoprotein. *J. Biol. Chem.* 278 36365–36372. 10.1074/jbc.M305449200 12869568

[B41] Thukkani A. K. Hsu F. F. Crowley J. R. Wysolmerski R. B. Albert C. J. Ford D. A. (2002). Reactive chlorinating species produced during neutrophil activation target tissue plasmalogens: production of the chemoattractant, 2-chlorohexadecanal. *J. Biol. Chem.* 277 3842–3849. 10.1074/jbc.M109489200 11724792

[B42] Thukkani A. K. Martinson B. D. Albert C. J. Vogler G. A. Ford D. A. (2005). Neutrophil-mediated accumulation of 2-clhda during myocardial infarction: 2-clhda-mediated myocardial injury. *Am. J. Physiol.* 288 H2955–H2964. 10.1152/ajpheart.00834.2004 15681699

[B43] Verheul H. M. W. Jorna A. S. Hoekman K. Broxterman H. J. Gebbink M. F. B. G. Pinedo H. M. (2000). Vascular endothelial growth factor–stimulated endothelial cells promote adhesion and activation of platelets. *Blood* 96 4216–4221. 11110694

[B44] Wang W. Y. Albert C. J. Ford D. A. (2014). Alpha-chlorofatty acid accumulates in activated monocytes and causes apoptosis through reactive oxygen species production and endoplasmic reticulum stress. *Arterioscler. Thromb. Vasc. Biol.* 34 526–532. 10.1161/ATVBAHA.113.302544 24371082 PMC3951512

[B45] Wildsmith K. R. Albert C. J. Anbukumar D. S. Ford D. A. (2006). Metabolism of myeloperoxidase-derived 2-chlorohexadecanal. *J. Biol. Chem.* 281 16849–16860. 16611638 10.1074/jbc.M602505200

[B46] Wu S. Zhou C. King J. A. Stevens T. (2014). A unique pulmonary microvascular endothelial cell niche revealed by weibel-palade bodies and griffonia simplicifolia. *Pulmon. Circ.* 4 110–115. 10.1086/674879 PMC4070765 25006426

[B47] Ysebaert D. K. De Greef K. E. Vercauteren S. R. Ghielli M. Verpooten G. A. Eyskens E. J. (2000). Identification and kinetics of leukocytes after severe ischaemia/reperfusion renal injury. *Nephrol. Dialysis Transpl.* 15 1562–1574. 10.1093/ndt/15.10.1562 11007823

[B48] Yu H. Wang M. Wang D. Kalogeris T. J. McHowat J. Ford D. A. (2018). Chlorinated lipids elicit inflammatory responses in vitro and in vivo. *Shock* 51 114–122. 10.1097/SHK.0000000000001112 PMC6070441 29394241

